# Link between Cell Junctions and Microtubule Cytoskeleton Is Critical for Epithelial Morphogenesis

**DOI:** 10.1371/journal.pbio.1002088

**Published:** 2015-03-12

**Authors:** Richard Robinson

**Affiliations:** Freelance Science Writer, Sherborn, Massachusetts, United States of America

Epithelial cells separate what’s inside us from what’s outside, forming the skin as well as the linings of the gut, lungs, kidneys, and other organs that communicate with the external world. Individual cells link up with their neighbors through a variety of specialized cell junctions, allowing them to control the passage of materials between inside and outside. That regulatory function is further enhanced by the polarized nature of most epithelial cells, in which the set of proteins localized to the apical pole, in contact with the external environment, differs dramatically from those localized to the basal pole, where the cell is anchored.

A ubiquitous type of linkage between epithelial cells is the adherens junction, in which transmembrane cadherin proteins in adjacent cells link to each other in the presence of calcium. Just within the membrane, cadherins link to catenins, which are in turn linked to the actin cytoskeleton. A small but growing body of work has also suggested that microtubules play critical roles at adherens junctions, but evidence for a direct linkage has been unclear. In a new study, Maria Gavilan, Marina Arjona, Rosa Rios, and colleagues show that a protein better known for its role at the centrosome is critical for connecting microtubules to adherens junctions through direct binding with alpha-catenin. Loss of that linkage disrupts apico-basal morphogenesis and prevents normal differentiation of epithelial cells.

The protein, called CAP350, binds directly to microtubules, and is known to localize at the centrosome. While some evidence has hinted that it may play a role in organizing the cytoskeleton at the membrane, most studies of the protein have been performed in cells that do not make adhesive contacts with adjacent cells. Using an anti-CAP350 antibody, the authors first showed that CAP350 was indeed found at the periphery, specifically co-localizing with alpha-catenin at adherens junctions. Intriguingly, the staining was strongest when the cell was fully polarized.

Disrupting adherens junctions, either through block of E-cadherin, loss of alpha-catenin, or chelation of calcium, depleted CAP350 peripherally but not at the centrosome; restoring calcium restored CAP350. Further experiments showed that CAP350 bound directly to alpha-catenin, and that without it, CAP350 could not be recruited to the junctions. CAP350’s ability to bind both microtubules and alpha-catenin suggests it acts as a critical link between the junction and the cytoskeleton.

Just how critical that link is became evident in examining the effect of CAP350 knock-down on the architecture of a type of model kidney epithelial cell called MDCKII (Fig. 1). In vivo, kidney epithelial cells are elongated into columns, which, when aligned and rolled into a tube, form the critical secretory tubules within the nephron. In cell culture, MDCKII cells instead normally form a hollow ball, called a cyst. But without CAP350, the microtubule network within the cells mimicked that of nonpolarized cells; the cells themselves were flattened, the network of adherens junctions was disrupted, and the number of cell clusters able to form well-developed cysts dropped dramatically. In a model of epithelial morphogenesis during embryo development, lack of CAP350 interrupted multiple aspects of cell migration and tissue formation.

**Fig 1:**
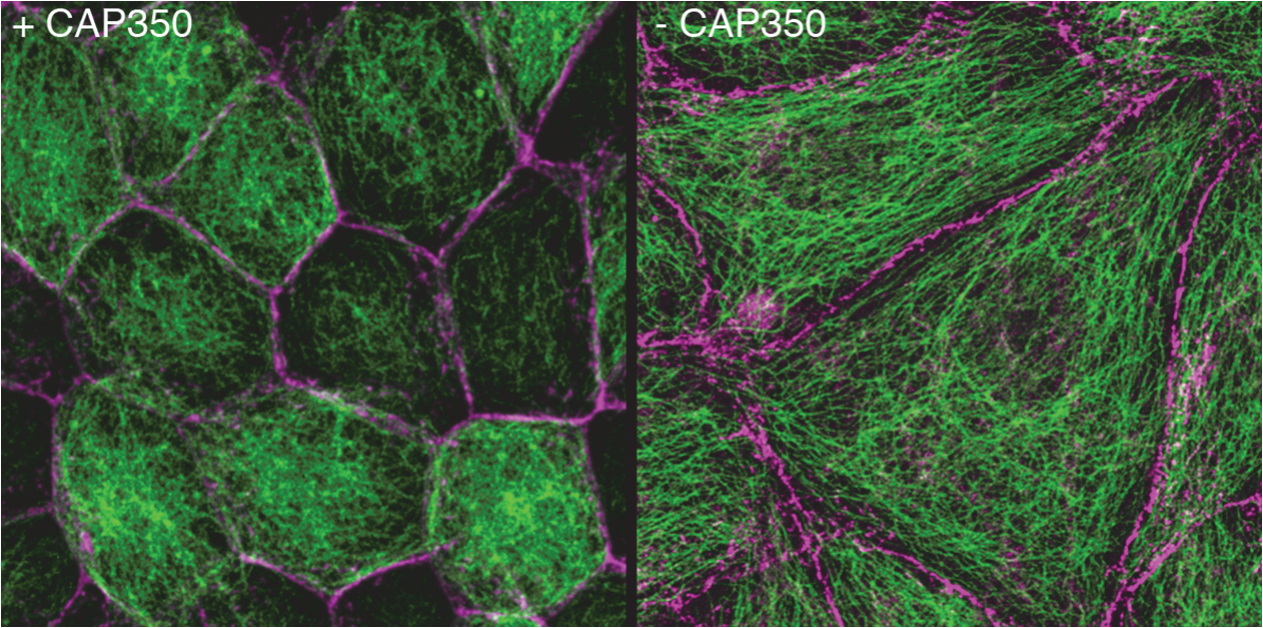
The loss of CAP350 impairs epithelial morphogenesis in kidney cells. Microtubule network, green; cell junctions, magenta.

This study highlights the importance of the microtubule cytoskeleton for morphogenesis of cell architecture, as well as the critical role played by cell junctions in shaping cell development. It may also have some direct practical applications, since failure of proper junction formation underlies several human diseases.

## References

[1] Gavilan MP, Arjona M, Zurbano A, Formstecher E, Martinez-Morales JR, Bornens M, et al. (2015) Alpha-catenin–Dependent Recruitment of the Centrosomal Protein CAP350 to Adherens Junctions Allows Epithelial Cells to Acquire a Columnar Shape. doi:10.1371/journal.pbio.1002087 10.1371/journal.pbio.1002087 PMC4357431 25764135

